# Self-Assembly of Human Serum Albumin: A Simplex Phenomenon

**DOI:** 10.3390/biom7030069

**Published:** 2017-09-20

**Authors:** Garima Thakur, Kovur Prashanthi, Keren Jiang, Thomas Thundat

**Affiliations:** Department of Chemical and Materials Engineering, University of Alberta, Edmonton, AB T6G-1H9, Canada; kovur@ualberta.ca (K.P.); kjiang@ualberta.ca (K.J.); thundat@ualberta.ca (T.T.)

**Keywords:** protein self-assembly, Pascal’s triangle, *n*-simplex, geometric pattern, thermodynamics, kinetics

## Abstract

Spontaneous self-assemblies of biomolecules can generate geometrical patterns. Our findings provide an insight into the mechanism of self-assembled ring pattern generation by human serum albumin (HSA). The self-assembly is a process guided by kinetic and thermodynamic parameters. The generated protein ring patterns display a behavior which is geometrically related to a *n*-simplex model and is explained through thermodynamics and chemical kinetics.

## 1. Introduction

Nature elegantly produces several self-assemblies of complex biological structures by hierarchical growth of nanometer scale building blocks. Some of the outstanding and simple examples include the folding of peptide chains into proteins, self-assembly of proteins into shells of viruses, spontaneous assembly of cells into tissues, and nucleotides into a complex DNA structure. Molecular self-assembly is a smart process because of its simplicity and the potential efficiency for designing structures at small scale in spontaneous assembly of molecules [1,2,3,4,5,6]. Essentially, non-covalent interactions pave the way to subtle transitions in self-assembled arrangements to form smart materials using protein/peptides [7]. Spatial properties or topology of the monomeric units are quintessential in determining the shape, configuration, and properties of the self-assembled material. Depending upon the amino acid sequence or structure of the peptide or protein, various types of self-assemblies can be produced. Some of the assemblies include, but are not limited to, tubular, ring-shaped, cage-like, polyhedral, combined or complex structures, and one-dimensional fibrous assemblies [8]. Predominantly in the search for new and cheaper materials for controlled fabrication, the use of peptides and proteins has emerged tremendously in last decade. Specifically, for biomedical diagnostic applications, precise control of the orientation and position of assemblies is a key factor [9]. Enhanced sensitivity of 3-D diagnostic assays may be accomplished using self-assembly of nanoparticles/nanostructures in conjugation with self-assembled biomolecules/proteins [8,9]. Ease of structural manipulation, the symmetry, and chemical/physical properties of proteins make protein assemblies useful in various nanotechnology applications, for example, arrangement of nanoparticles in multidimensional patterns and generating new functional materials with exceptional applications [6,7,8,9].

Several approaches have been employed to understand the driving forces and mechanisms of self-assembly. For example, electrostatic interactions [10], gravity [11], magnetic force [12], and entropy [13] have been explained using simulations and through mathematical models. One of the characteristic features of formation of self-assembled structures is the minimization of the free energy of formation. In literature, self-assembled patterns [14,15], ordered arrays of polymers, co-polymers [16], polymer brushes [17], nanoparticles on surfaces [18,19], and growth of mesoporous silica [20] have been shown to be a direct consequence of minimization of free energy of formation of structures or assemblies. There are very few artificially or synthetically organized assemblies which resemble natural geometric patterns. Indeed, impressive experimental advances have been achieved in the realms of self-assembly, however, the phenomenon is not understood theoretically. In our previous work, we have reported the self-assembly of human serum albumin (HSA) on a pre-patterned gold surface using carboxylated polyethylene glycol (PEG_12_-CL). To simplify we will address the pre-patterned surface as PEG- islands or PEG in the manuscript. Ring structures are formed with the nucleus in the center and beads of HSA protein aggregates surround the nucleus in concentric rings [21,22]. These organized assemblies have striking resemblance to Pascal’s *n*-simplex geometry. To the best of our knowledge, most of the biomolecular self-assemblies are not seen through the perspective of mathematical geometry. 

An understanding into the mode of formation of the ring structures has been obtained using scanning electron microscopy (SEM), and atomic force microscopy (AFM). These techniques give substantial information regarding the mechanism of formation of self-assembled patterns. Here, we propose the mechanism of formation of geometric patterns of HSA in terms of a mathematical geometric model of *n*-simplex and its bridging towards the thermodynamics and kinetics of self-assembly. The main purpose of this paper is to give an insight into the mechanism from the perspective of mathematical geometric design, and how it may be related to chemical thermodynamics and kinetics.

## 2. Results

### 2.1. Experimental Results

**Figure 1:**
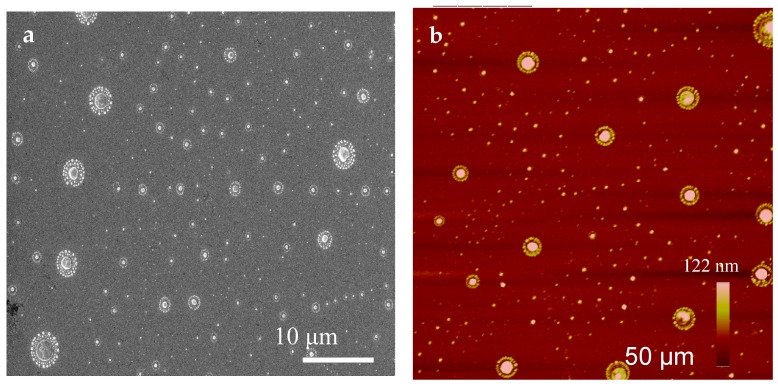
Protein self-assembly: (**a**) scanning electron microscopy (SEM) and (**b**) atomic force microscopy (AFM) images of discrete protein ring patterns.

**Figure 2:**
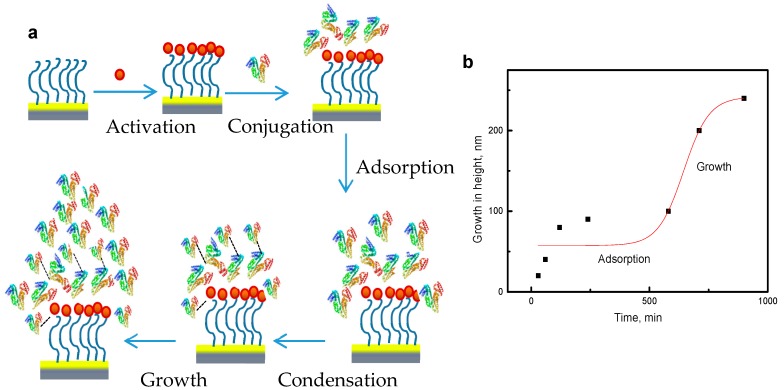
Mechanism of pattern formation: (**a**) scheme of protein self-assembly and (**b**) growth in height of the self-assembly with time.

AFM and SEM images of the self-assembled ring patterns are presented in Figure 1. In summary, for directed self-assembly, a bio-conjugation approach to form protein ring patterns around PEG-islands was used on gold-coated silicon substrates. Distinguished features are seen in the images, showing the nucleus and the beads around the nucleus. The nucleus is the PEG-island and the beads formed around it or on top of it consist of the protein [21]. The mechanism of formation of the ring patterns on the pre-patterned PEG surface consists of several steps that may be described as conjugation, adsorption, condensation, and growth. A schematic of ring pattern formation is shown in Figure 2.

The ring patterns formed consist of a nucleus in the center and concentric rings of beads around the nucleus. Figure 2a illustrates the pre-patterned substrate, which means the surface was first covered with a PEG self-assembled monolayer (SAM). The substrate covered with PEG was then activated using 1-ethyl-3-(3dimethylaminopropyl)carbodiimide hydrochloride (EDC) and *N*-hydroxysulfosuccinimide (NHS-sulfo) in phosphate buffer saline (PBS) at pH 7.4. The next step is conjugation of the protein HSA to the activated surface. During the incubation time, adsorption of the protein takes place, followed by condensation and growth of the pattern. The incubation time was around 15 hours and the growth in self-assembled patterns was a slow process. The height of the structures increased with time from 20 to 240 nm [21]. The properties and formation of these structures are elaborately described in our previous work [21]. The growth in height of the self-assembly process was sigmoidal and follows the Boltzmann law from adsorption to growth of the patterns (Figure 2b). The growth of the ring pattern is guided by thermodynamics and kinetics of self-assembly, which we will discuss in proceeding sections.

### 2.2. Pattern Similarity to Geometric n-Simplex

**Figure 3:**
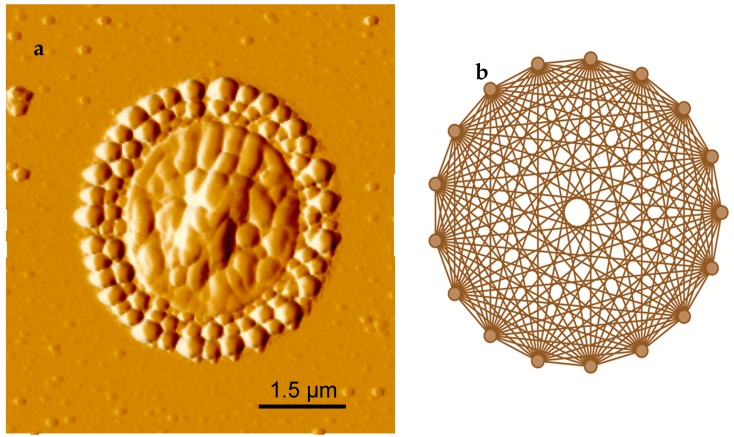
Comparison of the ring pattern to geometric design: (**a**) AFM image of a single protein ring pattern and (**b**) geometric *n*-simplex.

First, let us check the symmetry of the ring, carefully looking at the pattern formation from the HSA on PEG coated surface, it reminds one of Pascal’s *n*-simplex patterns in mathematical terms (Figure 3). To understand the radial symmetry of this ring pattern resembling the simplex geometry we need to go back and check what Pascal’s *n*-simplex is. Let us consider an example of Pascal’s triangle, which explains the multiplicity of a result of a series of binary events [23].

**Figure 4:**
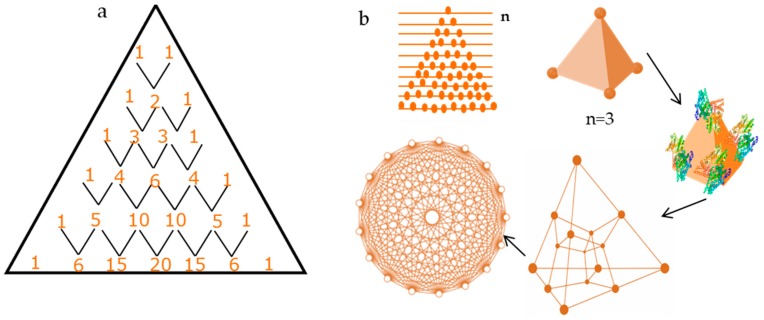
Diagram showing connection of Pascal’s triangle to geometric design: (**a**) illustration of Pascal’s Triangle and (**b**) Sketch of Pascal’s triangle and *n*-simplex when *n* = 3, representing a tetrahedron structure, when tetrahedron structures join together symmetrically into a 120-cell unit it gives a 2-D projection or symmetric unit which resembles the ring pattern.

Figure 4a represents Pascal’s triangle. In mathematics, it represents a triangular array of binomial numbers. In higher dimensions, the general term used is Pascal’s simplices, which is an extension of Pascal’s triangle into arbitrary dimensions (*n*). Geometrically, Pascal’s *n*-simplex can be represented by the following equation that consists of coefficients of multinomial expansion of a polynomial *n* raised to the power m. It means that *m*th component of the *n*-simplex has (*n* − 1) dimensions [23,24,25].

(1) $$\wedge_{m}^{n}=\Delta_{m}^{n-1}$$

(2) $${\left(\overset{n}{\underset{i=1}{\sum }}x_{i}\right)}^{m}=\underset{\sum_{i=1}^{n}k_{i}=m}{\sum }\left(\begin{array}{c} m\\ k_{1},\;k_{2},\ldots k_{n} \end{array}\right)\overset{n}{\underset{i=1}{\prod }}x_{i}^{k_{i}}$$

where $k_{1},\;k_{2},\ldots k_{m}\in \;{\mathbb{N}}_{0,};i,\;m\;\in \mathbb{N}$.

The terms involved in Pascal’s simplex can be represented using symmetric (*n* × *n*) Pascal’s simplex matrix, for example simplex with *n* = 4 can be denoted as

(3) $$\left[\begin{array}{cccc} 1 & 1 & 1 & 1\\ 1 & 2 & 3 & 4\\ 1 & 3 & 6 & 10\\ 1 & 4 & 10 & 20 \end{array}\right]=\exp\left[\begin{array}{cccc} 1 & 0 & 0 & 0\\ 1 & 1 & 0 & 0\\ 1 & 2 & 1 & 0\\ 1 & 3 & 3 & 1 \end{array}\right]\exp\left[\begin{array}{cccc} 1 & 1 & 1 & 1\\ 0 & 1 & 2 & 3\\ 0 & 0 & 1 & 3\\ 0 & 0 & 0 & 1 \end{array}\right]$$

Early pioneering work on quantification of shape variation in biology was done by the zoologist D’Acry Thompson [26]. Quantification of shape variation is of interest to morphometricians and many statistical methods have been evolved by mathematicians [27]. The process of the directed self-assembled growth of protein may be broken down into two parts if we consider it from a geometric perspective: (i) To obtain a larger set of the system (pattern), one must determine the subset of the smallest unit cell involved. (ii) Symmetric and non-symmetric conditions which may result in the generation of a pattern from unit cells would determine the stability of the pattern. Figure 4b shows the case of Pascal’s triangle and *n*-simplex (with *n* = 3), which is a geometric representation of a tetrahedron structure. In 2-D orthogonal projections, the geometric pattern formed has radial symmetry. It would also be true to say that for the construction of a larger pattern, there would be smaller unit cells involved.

The stability in physical terms for any pattern or any structure or shape is a consequence of thermodynamic stability, and the growth depends on the kinetics of the involved reactions. In the next section, we will try to uncover how the thermodynamics and kinetics may be related to mathematical geometry.

### 2.3. Thermodynamics of Pattern Formation

The fundamental question which arises is: why does this pattern form? To understand the events leading to pattern formation, we will explore the process in terms of statistical thermodynamics. Self-assembly of biomolecules is a spontaneous natural process. Once initiated, it might lead to a final molecular assembly without outside intervention. The final state of self-assembly would contain the particles/molecules/biomolecules or energy in the most widely dispersed manner, leading to state of equilibrium. In the case of bio-conjugation on the substrate, the driving force is the strong binding interaction of biomolecules on PEG self-assembled monolayer with active sites. This can be related to the first step of conjugation of protein molecules. In thermodynamic terms, it means that this spontaneous change of a statistically significant number of biomolecules (possessing thermal energy) is driven by the tendency of the biomolecules to spread in as many microstates as thermally accessible in the system and its surroundings [28,29].

Let us assume there are *M* numbers of binding sites present on a substrate. Consider that there are *N^a^* free protein monomers and *N^b^* aggregated protein molecules present in the solution with concentration *C* and total volume *V*. The aggregated proteins may contain different numbers of subunits (*x*). The total number of proteins in solution can be defined as:

*N^a^* + *x N^b^* = *N* (4)

Instead of considering different conformations of protein molecules, let us define them in terms of different energy states. Let us consider the case of HSA protein, since these different configurations of the protein in solution with a constant volume *V* will have a total energy *U*. The multiplicity of the system can be defined in terms of the number of equivalent energy rearrangements of the protein molecules in the system. Let us consider that the total energy (*U_total_*) of the system is constant.

(5) $$U_{total}=\underset{i}{\sum }N_{i}U_{i}$$

where *N_i_* is number of protein molecules in *i*th energy state and *U_i_* is the energy of the *i*th state.

The entropy of the system can be described statistically in terms of multiplicity (*W*) relating to number of events or arrangements and configurations (microstates) [28]. The distribution of biomolecules is dependent on finding the configuration of particles (biomolecules) in the system that yields the maximum value of *W*, bound by the constraint of the total number of molecules (*N*) and the constant internal energy (*U_total_*) [30]. As the system becomes more complicated, a statistical approach brings a fresh way to describe entropy. The conformational rigidity of the protein on a solid support is a consequence of the thermodynamic equilibrium stability of the protein, minimizing the entropy gain, and leading to an unfavorable conformation [31]. Let us consider PEG as a single point attachment ligand, which can bind to a free, negatively charged protein present as a receptor in the solution. Two events can transpire; either binding of monomer or binding of aggregates to the ligand. If there are *M* ligand attachment sites present on the substrate, the number of positive outcomes or number of ways of arranging the protein molecules can be calculated as:

(6) $$W=\frac{\;M!}{N!\left(M-N\right)!}=\left(\begin{array}{c} m\\ n \end{array}\right)$$

The results are represented by Pascal’s triangle, where the property of binomial coefficients, which is true for any *m* and *n* combinations, can also be represented by the following equation [32]:

(7) $$\overset{j}{\underset{m=0}{\sum }}B\;\left(m,\;n\right)=B\left(m+1,\;n+1\right)$$

As the number of particles in the system increases, there is an interesting property that appears; namely, there are (*n* − 1) hyperplanes for the microstates of *n*-particles in the system [32]. Since there are large numbers of protein molecules present in the solution, Stirling’s approximation can be applied to the system and can be represented by the formula

(8) $$\ln W\left(M,N\right)=Nln\frac{M}{N}$$

The entropy (*S*) of the system can be linked to the multiplicity (*W*) and Boltzmann constant (*k_B_*) of the system, as

*S* = *k_B_* ln*W* (9)

or

(10) $$\;S=\;-Nk_{B}\overset{t}{\underset{i=1}{\sum }}p_{i\;}lnp_{i}$$

where *p_i_* is the fraction of protein molecules in the ith energy level, *p_i_* = *N_i_*/*N* and *N* is the total number of protein molecules [28,29,32]. The total partition function of the system can be represented as a combination of the distribution of different molecules in different energy levels.

(11) $$Q_{total}=\;\underset{i}{\prod }\frac{{\left(Q_{A\prime }\;V\right)}^{N^{a}}\;{\left(Q_{B\prime }\;V\right)}^{N^{b}}}{N^{a\;}!\;N^{b}!}$$

where *Q_A′_* and *Q_B′_* correspond to the partition functions of monomers and micelles, respectively.

(12) $$Q_{A}=\;\overset{n}{\underset{i=1}{\sum }}e^{-\beta \varepsilon_{i}}$$

(13) $$Q_{A\prime }=\int^{\text{​}}\Lambda_{a}\;d\left[q\right]e^{-U\left(q\right)/Tk_{B}}$$

The partition function *Q_A_* is known to be exponential in nature and represents the Boltzmann distribution of molecules over different energy levels, which can be expanded for protein molecules, where *Λ_a_* is volume of the coordinate space defining the protein monomer and [*q*] denotes the set of degrees of freedom of all the atoms making up the molecule, and *U*(*q*) is the internal energy of the system [33]. For a system of *N* molecules to generate a pattern which is governed by thermodynamic stability, the entropy of the system plays an important role, given by Equations (9) and (10). According to Equation (9), “*W*” represents the number of ways of distributing the molecules to achieve a specific macroscopic state. To generate a specific pattern which resembles a Pascal’s simplex pattern, in our case, the most probable macroscopic state will evolve with the highest value of *W* which could be represented by Pascal’s simplex Equation (3).

Let us consider that the entropy distribution of protein molecules results in simplex pattern formation. The change in entropy of a large number of protein molecules results in pattern formation governed by the mathematical rules of Pascal’s simplex. According to the concept of Pascal’s distribution in statistical thermodynamics for n number of particles, there are (*n* – 1) hyperplanes [32]. For the distribution of protein molecules in 3-D space (substrate with x, y, and z coordinates), a minimum of 4 protein molecules are required. In three dimensions, the one that stands out would be the probability distribution which should resemble the Pascal’s triangle, and the most predictable one would be a 3-simplex. Let us write a lower triangular Pascal’s matrix (*n* = 4) leading to *n*-simplex ($\Delta^{n-1}$)

(14) $$\left(\begin{array}{cccc} 1 & 0 & 0 & 0\\ 1 & 1 & 0 & 0\\ 1 & 2 & 1 & 0\\ 1 & 3 & 3 & 1 \end{array}\right)\begin{array}{c} i_{1}\\ i_{2}\\ i_{3}\\ i_{4} \end{array}$$

The left hand side of the (4 × 4) matrix (in Equation (14)) corresponds to a tetrahedron, which is a 3-dimensional unit. The right hand side of the matrix represents energy levels (*i*_1_ to *i*_4_). The most consistent microstates leading to a particular microstate, i.e., the simplex phenomenon in this case could be represented by a 4 × 4 Pascal’s matrix. Hence, a steady state at equilibrium resulting in a thermodynamically stable macrostate is subject to the constraints of a constant concentration of protein molecules and a constant internal energy [28]. The number of microstates possible through this outcome is fifteen. However, the thermodynamic growth of the pattern follows an intriguing connection to the number theory represented by the simplex matrix, which is related to Boltzmann distribution. This matrix provides valuable information about the protein molecules in terms of energy and reaction dynamics. Conversely, in a realistic model with degenerate energy levels having unequal spacing, the energy distribution becomes complicated. To follow the simplex pattern, the microstates are subject to the constraints in Equation (12), where the energy levels are multiples of the constant *ε_i_* and allowed multiples are subject to take on integer values of 0, 3, 8, 15, …, *m*, for *n* number of energy levels to follow the Pascal’s distribution [28,29,30,31,32,33]. When there is perturbation from symmetry, only the magnitude of *β* may be changed in Equation (12), and there is a reduction in the number of microstates possible for the self-assembly. The distribution of different molecules in turn can be determined by the minimum of the total free energy of the system.

(15) $$G_{total}\;=\;\frac{k_{B}T\;ln\;Q_{total}}{V}$$

The same reasoning can be applied for the aggregated proteins with *x* (where *x* > 1) subunits with the condition that the number of microstates stays same in each energy level, obeying the rule of Pascal’s triangle. However, in this particular case of HSA protein, it was observed that there was no change in the solution conformation of the protein, and the protein was mostly found to be in its monomeric form in solution over a period of time. These results show that the entropy change leading to tetrahedron units of the protein might be the most stable steady state or intermediate structure thermodynamically in 3-D space. The average height of the small, circular patterns around the big nucleus is 80 nm (Figure 2), which corresponds to approximately 15 HSA protein monomers (radius of HSA monomer in solution being 27.4 ± 0.35 Å) [34]. However, a point to be noted here is that the HSA molecules were present in a NaCl solution, whereas, in the present case, PBS buffer was used.

### 2.4. Chemical Kinetics of Pattern Formation

Let us consider the kinematics of the reaction process, which may play an important part in self-assembly, and encompasses chemical kinetics, motion of molecules, and mathematics. To analyze the dynamic behavior of the system, it is useful to begin at the end. We observed an interesting behavior of pattern formation on the gold substrate by HSA. There are generally multiple or infinitesimal reactions proceeding simultaneously in any complex biological system. Let us assume *N_i_* number of protein molecules (state variables, in *i*th energy level) participating in the binding process in self-assembly, which proceeds by *j* number of pathways or reactions. If we consider that the Pascal’s matrix also holds good for the stoichiometry of the reaction, let us see the probable reaction pathways with which the reaction mechanism can be predicted. According to Poincare–Benedixson theorem [35]: In a 2-D system (i.e., 2 concentration variables) which is confined to a finite region of concentration space (e.g., because of stoichiometry and mass conservation), a steady state must ultimately be reached, or the system will oscillate periodically. Consider a model with 3 independent concentration variables, *α*, *β*, and *γ*, participating in a chemical reaction, whose time variables are represented by three functions *f*, *g*, and *h*, respectively.

(16) $$A\overset{k_{1}}{↔}B\left(\alpha \right)\overset{k_{2}}{↔}C\left(\beta \right)\overset{k_{3}}{↔}P\left(\gamma \right)$$

(17) dα/dt=f(α, β,γ)

(18) dβ/dt=g(α, β,γ)

(19) dγ/dt=h(α, β,γ)

Hence, the Jacobian matrix for the reaction at steady state can be represented as

(20) $$J=\left[\begin{array}{ccc} \partial f/\partial \alpha & \partial f/\partial \beta & \partial f/\partial \gamma \\ \partial g/\partial \alpha & \partial g/\partial \beta & \partial g/\partial \gamma \\ \partial h/\partial \alpha & \partial h/\partial \beta & \partial h/\partial \gamma \end{array}\right]$$

The results can be represented in a compact form as (**J** − *λ***I**) **C** = 0, where **J** is the Jacobian matrix, **I** is the identity matrix, **C** is the vector of coefficients, and *λ* is the eigenvalue [36]. Such a system may grow or decay depending upon the stability of the system [35,37]. If the eigenvalue *λ* has a positive real part, the solution will grow exponentially and the steady state is unstable. This implies that the sum of the diagonal elements of the Jacobian matrix is a real number or *tr*(**J**) > 0. *n* × *n* Jacobian matrix elements associated with steady state can be defined as **J*_ij_*** = ($\partial f_{i}/\partial x_{j}{)}_{\mathrm{ss}}$.

Considering that the pattern grows symmetrically, represented by a Pascal’s simplex, with the sum of the diagonal elements represented by [24],

(21) $$tr\left(Sn\right)\;=\;\overset{n}{\underset{i=1}{\sum }}\frac{\left[2\left(i-1\right)\right]!}{{\left(\left[i-1\right]\;!\right)}^{2}}$$

Hence, for the growth of the pattern exponentially away from the steady state, *tr*(**J**) >0 ≈ *tr*(*Sn*). Alternatively, we may consider Lyapunov exponents, the quantities representing the attracting trajectories of the Jacobian eigenvalues that characterize the steady state. In a dynamic system, the Lyapunov exponent may be described by the average rate of growth of *n*-dimensional volume around the evolving attractor in the *n*-dimensional phase space [38,39]. The number of linearly independent chemical reactions shows the dimensionality of the reaction in the reaction space. This implies that the number of chemical species essentially involved in the reaction is more than the number of linearly independent reactions. This is true for our matrix relation where the number of species is more than the dimension of the matrix i.e., 3. This also confirms the fact that the self-assembly starts at the nanoscale and eventually turns into a macro-scale event over a period of time determined by the reaction rate. Nonetheless, protein–protein association requires a transition state for the association mechanism. In the case of no large conformational changes being observed in the protein, the association rate advances toward the diffusion-limited rate (*k* ≤ *k_diff_*) [38].

*k_a_* = *k_B_T*/*h* exp(−ΔG^++^/*k_B_T*) (22)

where *k_a_* is the rate of association of the transition state, *h* is Planck’s constant, Δ*G*^++^ is Gibb’s free energy change for the transition state, and *T* is the temperature. If we assume the tetrahedron structure is a transition state intermediate, we can predict the pattern formation using Equation (20)**.** Let us use a simple method to understand the kinetics, assuming a reaction pathway:

(23) $$A+A_{x\;}\overset{k_{1}}{\to }P$$

where A is a protein molecule and A*_x_* is *x* units of protein molecules associating to give the final product P, and the kinetic rate constant is *k*_1_ for the association. If there is a dominant intermediate state (transition state) AA*_x_*^++^ formed in the reaction pathway, and the associated rates would be shown as:

(24) $$A+A_{x}\;\overset{K^{++}}{↔}{\mathrm{AA}}_{x}\overset{k^{++}}{\to }P$$

(25) $$K^{++}=\;\frac{\left[{A\;A}_{x}^{++}\right]}{\left[A\right]\;\left[A_{x}\right]}=\exp\left(\frac{-\Delta G^{++}}{RT}\right);\;k^{++}=\;\frac{k_{B}T}{h}$$

Rate of decomposition of [AA*_x_*]

(26) $$\frac{-d\;\left[{\mathrm{AA}}_{x}^{++}\right]}{dt}\;=-k^{++}\left[{\mathrm{AA}}_{x}^{++}\right]$$

(27) $$=\left[A\right]\frac{k_{B}T}{h}\exp\left(\frac{-\Delta G^{++}}{RT}\right)$$

(28) $$k1=\frac{k_{B}T}{h}\exp\left(\frac{-\Delta G^{++}}{RT}\right)$$

(29) $$A+A_{x}\overset{k_{1}}{\underset{k_{-1}}{⇌}}{\mathrm{AA}}_{x}$$

where,

(30) $$k_{1}=\frac{kT}{h}e^{-\Delta G_{1}^{++}/RT};\;k_{-1}=\frac{kT}{h}e^{-\Delta G_{-1}^{++}/RT}$$

(31) $$K_{eq}=\;\frac{k_{1}}{k_{-1}}=\exp\left(-\frac{\left(\Delta G_{1}^{++}-\Delta G_{-1}^{++}\right)}{RT}\right)$$

(32) $$\Delta G^{++}=\Delta H^{++}-T\;\Delta S^{++}$$

(33) $$k=\;\frac{k_{B\;}T}{h}\exp\left(\frac{\Delta S^{++}}{R}\right)\exp\left(-\frac{\Delta H^{++}}{R}\right)$$

## 3. Discussion

Carefully analyzing the rate Equation (33), which shows the relationship between the thermodynamic terms and the kinetics of reaction, and if the rate of reaction *k* determines the growth of the pattern formation, which can be represented by Pascal’s simplex matrix, then the associated pattern can be seen in mathematical terms, as shown in Equation (3). This indicates that the entropy and enthalpy of a reaction may be represented by a symmetric Pascal’s matrix. Certainly, there would always be some constraints which may allow the randomness in the system and perturb the perfect geometry. One of constraints is that there must be closely packed active sites (PEG islands) which help in driving the reaction. A second constraint would be the perturbation of charge that would create disorder and change in the orientation from perfect geometry, as is also seen using some SEM and AFM images of the patterns. Another factor which would contribute to the geometry is change in the structure of the protein over time, which was not observed in case of HSA. Structural changes were checked using dynamic light scattering (DLS) and circular dichroism (CD) measurements. More details about these experiments are provided in Supplementary Material. 

The entropy constrains of the reaction pathway, which is essentially leading towards the formation of patterns, is linked to the binding energy of the reaction. Anchoring of the protein on the support has an effect on the hydration of the protein molecule. Progressive attachment of the protein on the support can change the conformation of the protein. To remain in the native state, the protein’s attachment on the surface must minimize the entropy gain [31]. Binding of the protein on the solid support may change the multiplicity of the system, as the energy is being transferred from the solution to the substrate. Let us consider the binding energy of a protein monomer to the ligand on the surface as *Υ_a_*, and the binding free energy of aggregates on the protein *Υ_b_* as:

(34) $$\Upsilon_{a}\;=\;-T\Delta S_{a}\;–\;\Delta \varepsilon_{a}\;+\;p\Delta V_{a}$$

(35) $$\Upsilon {\;}_{b}=\;-T\Delta S_{b}\;–\;\Delta \varepsilon_{b}\;+\;p\Delta V_{b}$$

where the change in entropy of a monomer is given by Δ*S_a_* and for the aggregate is given by Δ*S_b_*, as a result of loss of configurational freedom due to rigidity on the substrate. Δ*ε_a_*/Δ*ε_b_* represents a change in the internal energy of the protein/aggregates due to intra-molecular and inter-molecular interactions arising from interaction of a protein monomer and the ligand on the substrate due to various interactions, namely [38]: (i) van der Walls interactions, due to interaction between different atoms, (ii) solvation term, due to difference in solvation energy of polar and non-polar groups during binding on the substrate, (iii) hydrogen bond interactions arising due to differences in the free energy of formation between intra-molecular and inter-molecular H-bond interactions (with water), (iv) water bridge term, contributing towards extra stabilization of protein molecules because of more than one hydrogen bond, (v) electrostatic effects, including the contribution of charged groups, and (vi) entropy changes from backbone and side chains. Δ*V_a_*/Δ*V_b_* represents the change in volume of the monomer/aggregates upon binding to the substrate ligand. 

After the binding of protein on the charged ligand surface, the adsorption of protein molecules takes place. This can be explained by an electrical double layer, which makes an important contribution to the stability of the protein pattern formed [39]. Because of charge difference between the solid surface and the diffuse layer, there will be a charge gradient due to the movement of one relative to the other, thus creating potential difference. In the case of colloidal chemistry, the nonlinear Poisson–Boltzmann equation is derived in the context where the fixed charge does not arise from ionization or dissociation of a fixed number of groups of ions on a molecule or a membrane; but from adsorption of ions from salt that determine the potential [40,41,42]. There are two terms which affect the adsorption process; the first is a chemical work term and the second one is electrostatic work term. The chemical work term is steering the adsorption process, which persistently works until the chemical work gained by adsorption is precisely balanced by the electrostatic work spent to bring adsorbing ions to the surface. The chemical work per ion can be written as −*σϕ_o_*, where *σ* is the charge density of adsorbing ions, and *ϕ_o_*, is the final surface potential. This process takes place at constant potential and can be written in terms of free energy per unit surface area as [41,42]:

(36) $$\Delta G^{el}=\int^{\text{​}}\sigma \left(\phi \right)d\phi_{f}=\;-\sigma \phi_{o}+\int^{\text{​}}\phi_{f}\left(\sigma \right)d\sigma$$

In this expression, the second term represents the electrostatic work factor. This equation was first described by Levine for the adsorption process utilizing the osmotic pressure term [42].

(37) $$\Delta G^{el}=\;-\int^{\text{​}}\left(\frac{E.D.}{2}+\;\Delta \pi \right)dv\;$$

where *E.D.*/2 is the electrostatic stress and Δπ is the osmotic pressure of a mobile ion cloud. It is worth pointing out that the free energy for the process is negative. For macromolecules, such as proteins, equating Equations (36) and (37) and subtracting the chemical work term yields:

(38) $$\Delta G^{el}=\;\sigma \phi_{o}-\int^{\text{​}}\left(\frac{E.D.}{2}+\;\Delta \pi \right)dv\;$$

Electrostatic interactions play a fundamental role in essentially all reactions involving biomolecules. Electrostatics within the Poisson–Boltzmann equation for biomolecules provides a framework for reproducing, explaining, and predicting experimental observations [31]. However, the thermodynamics and kinetics play a vital role in the process, which includes the free energy term for protein molecules. One way of writing the infinitesimal Gibbs free energy change with respect to its natural variables (*P*,*T*) is given below

(39) $$dG=Vdp-SdT+\;\overset{k}{\underset{i=1}{\sum }}\mu_{i}\;dN_{i}$$

where the third term presents the chemical potential term for the ith particle, *μ* is chemical potential, and *N_i_* is the number of particles. At constant temperature and pressure, only the chemical potential term predominates. The electrostatic free energy term is shown by Equation (34), where the first term is chemical work per ion and the second term represents the electrostatic work factor. The total Gibbs free energy term is represented by Equation (15), where *Q_total_* is the partition function describing the canonical ensembles. In light of chemical potential and to simplify the calculations, we can use grand canonical partition ensemble (Ω), which includes the chemical potential [43].

(40) Ω=−kT ln∑microstateseμN−EkT

The grand canonical partition function over the sum of microstates can be written as

(41) $$X\left(\mu ,\;V,\;T\right)=\sum_{i}\exp(\mu N_{i}-E_{i})/kT$$

Upon inspecting Equations (15), (22), (32), (34), (35), and (38), it is clear that various arrangements for Δ*G* represent the number of ways of assembling the system to its final state. Henceforth, the perturbation in the pattern is possible depending upon the kind of ligand and protein used in the sample and the ionic atmosphere of the sample, i.e., the pH and/or buffer solutions used. To minimize distortion of the geometry, experimental error must be minimized. 

A system containing molecules can self-assemble if the constituents or the building blocks can equilibrate and become oriented in stable patterns over a period of time. Tailoring the functionalities of the building blocks could control the properties of the patterns. Modification of the constituents and controlling the time period of self-assembly can generate well-defined and ordered patterns. Self-assembly using polymers and/or biomaterials might result in striking arrays of structures. Self-assembly is a natural phenomenon in living systems, and patterning of biomaterials, such as proteins, has tremendous potential. Bio-conjugated protein micro-patterns, which are easy to prepare, could be used for various potential applications in the fields of biosensors, biomaterials, bio-micro-electro- mechanical-systems (bio-MEMS), cell adhesion, and bacterial growth.

## 4. Materials and Methods

### 4.1. Materials

Silicon substrate was obtained from Ted Pella Inc. (Redding, CA, USA). Chemicals, including NaCl, Na_2_PO_4_, EDC, NHS-sulfo and human serum albumin (30% in 0.85% sodium chloride) were purchased from Sigma Aldrich (Oakville, Ontario, CA). The ligands carboxy-PEG_12_-lipoamide (PEG_12_-CL), were purchased from Fisher Scientific (Rockford, IL, USA). Deionized (DI) water with resistivity of 18 MΩ·cm from Milli-Q-water purification system (EMD Millipore, Billerica, MA, USA) was used in all the experiments.

### 4.2. HSA Pattern Generation

Silicon substrate was cleaned in piranha (H_2_SO_4_:H_2_O_2_ (3:1)) and rinsed with plenty of water and coated with Ti/Au (5/50 nm) layers using e-beam evaporation. The substrate was cleaned again with piranha and ethanol before functionalization with 1 mM PEG_12_-CL in PBS buffer at pH 7.4. The chip was examined under a microscope for any scratches, and only smooth and clean surfaces were used for functionalization. Homogeneous SAM was formed after 4 h. The substrates were rinsed in buffer several times. After SAM formation, the carboxyl groups on PEG were activated using EDC (0.2 M) and NHS-sulfo (0.05 M) in MES buffer (pH 6) for 30 min with stirring. 0.2 mg/mL protein was functionalized on the activated substrate in PBS buffer at pH 7.4 for 15 h.

### 4.3. Atomic Force Microscopy

An AFM (Asylum Research, Santa Barbara, CA, USA) with tapping mode was used for the images of self-assembled patterns. A scanning rate of 1 Hz was used and SPIP 6.0.9 software (Image Metrology A/S, Hørsholm, Denmark) was used for the analysis of images.

### 4.4. Scanning Electron Microscopy

A Vega-3 scanning electron microscope was used for SEM (Tescan, Pleasanton, CA, USA) in High vacuum mode (pressure <3 × 10^−3^ Pa) with accelerating voltage between 200 eV and 30 keV for SEM imaging. Gel was rinsed with deionized water and mounted on SEM stubs.

### 4.5. Dynamic Light Scattering

DLS experiments were conducted on commercial apparatus ALV/CGS-3 compact Goniometer system (ALV GmbH, Langen, Germany) at an angle of 900. A JDS Uniphase 22 mW He–Ne laser, operating at wavelength 632.8 nm was used and was interfaced with a ALV-5000/EPP multi-tau digital correlator with 288 channels and a ALV/LSE-5003 light scattering electronics unit for stepper motor drive and limit switch control. Autocorrelation functions were collected 3 times for each solution and they were analyzed by the cumulants method and the CONTIN routine using the software provided by the manufacturer.

### 4.6. Circular Dichroism Spectropolarimetery

The CD spectra were measured on an OLIS DSM 17 Circular Dichroism instrument (OLIS Inc., Bogart, GA, USA). A quartz cell of 0.02 cm path length was used to contain the sample, and the spectra were recorded in the far-ultraviolet region with wavelength between 190 and 260 nm. The spectrum was recorded with five scan accumulations.

## 5. Conclusions

Self-assembly of biomolecules into symmetric patterns is valuable in the domain of biotechnology and biochemistry. In the present work, we have presented the mathematical relationship of Pascal’s simplex geometry which may be beneficial in understanding the thermodynamics and kinetics of self-assembly in the case of receptor–ligand-based chemistry. Thermodynamics and chemical kinetics play important roles in self-assembly, which leads to statistically favorable microstates of protein molecules, and in turn leading to *n*-simplex-like pattern formation. It can be understood from both results and theory that the most favorable states would lead to distribution of protein molecules in the format of Pascal’s triangle, which may lead to the formation of fundamental tetrahedron units as an intermediate state in the reaction pathway. Disorder in the resulting geometry is possible because of factors such as availability of binding sites, the environment of the reaction solution, such as pH, ionic strength, temperature, and charge on the molecules and the surface.

## Supplementary Materials

The following are available online at www.mdpi.com/2218-273X/7/3/69/s1, Figure S1: DLS measurements of HSA solution at different time periods, Figure S2: Trend of hydrodynamic radius vs. time period, Figure S3: CD measurement and comparison of HSA at time 0 h and 15 h.
