# Supplementary material for: Self-Assembly of Human Serum Albumin: A Simplex Phenomenon
Source: Biomolecules. 2017 Sep 20;7(3):69. doi: 10.3390/biom7030069 (PMC5618250; doi:10.3390/biom7030069)
Supplement: Supplementary file 1 [file biomolecules-07-00069-s001.pdf]

Supplementary Material

Self-Assembly of Human Serum Albumin: A Simplex Phenomenon

Garima Thakur *, Kovur Prashanthi, Keren Jiang and Thomas Thundat

Department of Chemical and Materials Engineering, University of Alberta, Edmonton T6G-1H9, Alberta

As described in the methods section of the manuscript, dynamic light scattering (DLS) experiments were conducted on commercial apparatus ALV/CGS-3 compact Goniometer system (ALV, GmbH, Germany) at an angle of 900. JDS Uniphase 22 mW He–Ne laser, operating at wavelength 632.8 nm. Autocorrelation functions were collected 3 times for each solution and they were analyzed by the cumulants method and the CONTIN routine using the software provided by the manufacturer. Figure S1 shows that the average hydrodynamic radius of human serum albumin molecules over a period of time stays at 3.9 ± 0.30 nm in the buffer solution. At time 0 hrs, around 99% of the sample has a radius of approximately 4.1 nm; at 15 hrs and 24 hrs, 76% and 71% of molecules exhibit radius of approximately 3.9 nm. Figure S2 shows the trend of radius over time.

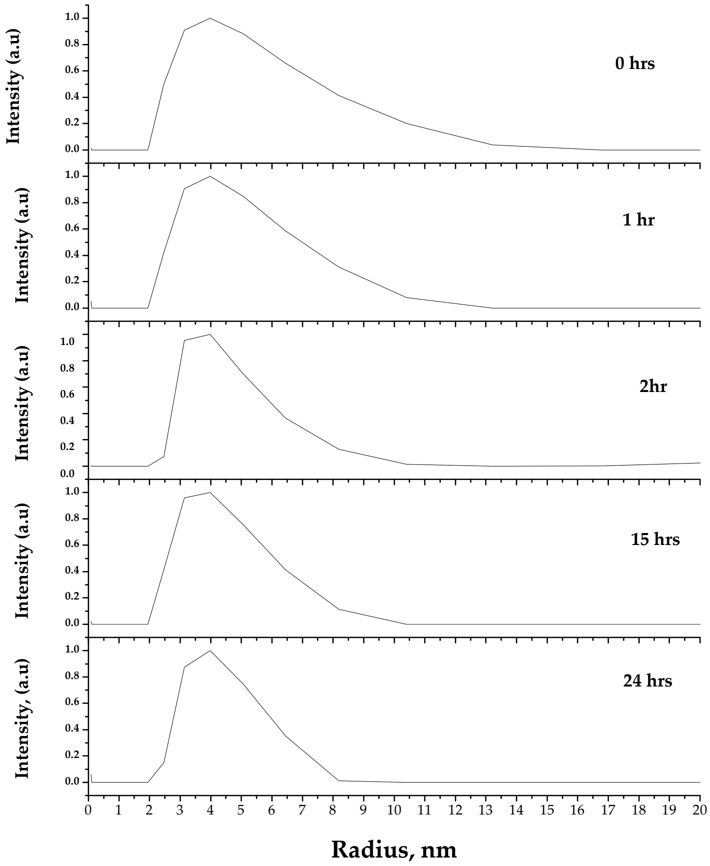

Figure S1 DLS measurements of HSA solution at different time periods.

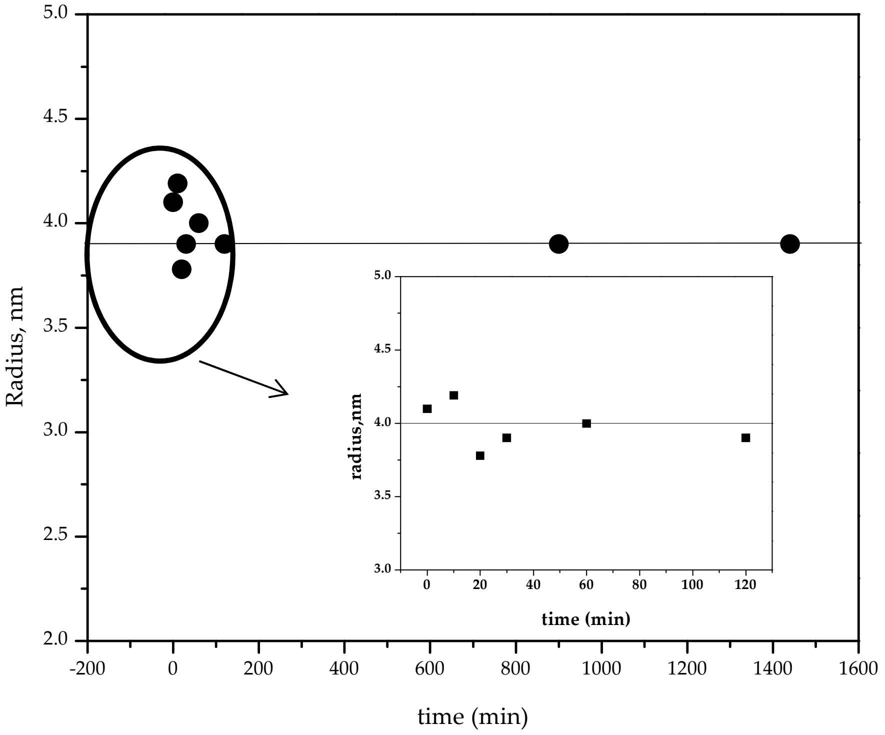

Figure S2. Trend of hydrodynamic radius vs. time period. Inset shows the range depicted by circle.

Figure S3 shows the CD spectra of a solution of HSA before and after incubation. The characteristic bands at 210 and 222 nm show that solution of HSA has the typical α-helical structure. Not much change in structure is evident after 15 hr. The initial concentration of HSA was 0.2mg/ml before incubation.

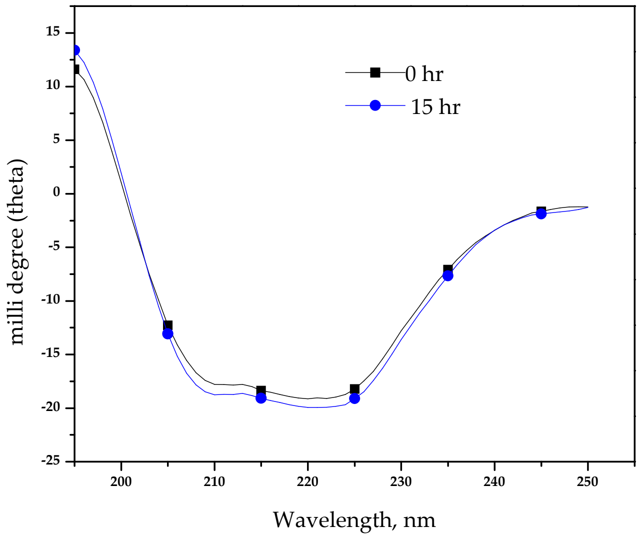

Figure S3. CD measurement and comparison of HSA at time 0 h and 15 h.
